# Efficacious biosorption of crystal violet pollutant dye from aqueous solutions via *Padina pavonica* derived alginate

**DOI:** 10.1038/s41598-025-09752-y

**Published:** 2025-07-12

**Authors:** Shimaa R. Dalal

**Affiliations:** Botany Department, Faculty of Science, Mansoura University, Mansoura, 35516 Egypt

**Keywords:** Crystal Violet dye, Alginate beads, *Padina Pavonica*, Biosorption, pH, Time, Concentration, Isotherm, Kinetic, FTIR and SEM, Biotechnology, Microbiology

## Abstract

Dyes are toxic, mutagenic, and hazardous to aquatic organisms. Calcium alginate beads derived from *Padina pavonica* were utilized for crystal violet (CV) bioremoval from water. They were examined using FTIR and SEM before and after CV biosorption, results demonstrated that the characteristics of alginate beads changed after CV biosorption. The bioremoval process of CV varies by environmental parameters including pH, incubation duration, alginate dosage, and initial CV concentration. These environmental factors influence on the CV biosorption process were investigated as one factor at a time. At pH 6, 70 mg/25 mL alginate dosage, and 2 mg L^− 1^ CV concentration after 5 h of incubation, the highest CV bioremoval efficiency was achieved. A correlation existed between the pseudo-second-order kinetic model and the kinetic assessments of the current results, which indicated chemisorption of CV on alginate beads. Moreover, the isotherms calculations revealed that the results were fit with Freundlich model, in which values of Freundlich constant (K_F_), correlation coefficient (R^2^) and n were 1.582, 0.988 and 2.378, respectively, indicating that CV particles are arranged as multilayers on alginate beads surface. Calcium alginate beads derived from *Padina pavonica* are promising in dye biosorption from aqueous solutions with no hazardous influence on the environment.

**Supplementary Information:**

The online version contains supplementary material available at 10.1038/s41598-025-09752-y.

## Introduction

A wide variety of manufacturing processes, including dyeing industry, fabric, paper, diet, printing, cosmetics, plastics and pharmaceutical industries, are responsible for the release of dyes into the water supply, making them one of the most significant environmental problems^1^. The existence of these compounds in bodies of water cannot be tolerated even at extremely low quantities because it is difficult to eradicate them due to the fact that they are very persistent and resistant^2^. Before the wastewater is released into the environment, it is necessary to remove the dyes that are present in the water since they are quite visible^3^. Moreover, due to the lower light penetration, aquatic life’s photosynthetic process suffer considerably^4^. Dyes are difficult to recover from dye wastewater due to high water solubility^5^. Moreover, The vast majority of inexpensive dyes are highly resistant to bioremediation and difficult to remove from aquatic solutions^6^. The visibility of textile effluent color removal has garnered attention more than its possible toxicity^7^.

Crystal violet, (which belongs to the triphenylmethane group), a textile and paper dye, is also found in navy blue and black inks for printing, ball-point pens, and inkjet printers. Crystal violet has many uses beyond just coloring. It’s an additive to poultry feed that stops the spread of fungus, intestinal parasites, and molds^8^. In some cases, it is used to color a wide range of products, including fertilizer, antifreeze, detergent, leather, and veterinary drugs^9^. Additionally, it is applied as a biological dye if necessary for the purpose of identifying fingerprints that include blood^10^. It is not safe for human consumption and has the potential to cause cancer when inhaled or swallowed^10^, moreover, it is teratogenic, and cause mitotic toxicity^11^. Therefore, before discharge into the environment, the removal of crystal violet from wastewater is urgent.

Biological, chemical, and physical methods have traditionally been used to remove dyes, degrade contaminants, or remediate them. Secondary water contamination can occur when chemical oxidation or coagulation is employed to treat wastewater that contains dyes, as these processes may introduce additional chemical substances^12^. On the other hand, physical remediation methods such as ozonation, irradiation, sludge production, membrane filtration, ion exchange electrochemical destruction, light degradation, chemo-oxidation, reverse osmosis, biosorption, ozonation^13^ and precipitation can be costly and produce undesirable by-products^14^. One common application of adsorption is the dyes removal from solutions in water. Although activated carbon is a widely used adsorbent, its regeneration is challenging, and equilibrium limits its performance^15^.

The utilization of living microorganisms, such as bacteria, yeasts, and fungi, as biosorbents comes under the category of biological treatment. It has been demonstrated that marine algae can be used effectively as biosorbents to remove a wide variety of harmful chemicals^16^. Biosorption offers a viable, economical, environmentally benign, and successful way to treat contaminated wastewater and remove heavy metals and textile dyes^17^. It has been reported that dangerous azo dyes can be effectively removed by using microorganisms or their enzymes^16^. Because of their surface properties, high affinity for binding metals, affordability, algae are thought to be efficient biosorbents for the metal biosorption from water. Additionally, the fact that they have a large number of active binding sites, such as hydroxyl, amino, and carboxylic groups, on their surface, makes them one of the most crucial methods employed in bioremediation by various processes, comprising complexation, ion exchange, and electrostatic forces^18^. Algal feeding demands are extremely minimal and do not produce hazardous chemicals, hence there is also very little need for minor hazardous chemicals^18^. Although active carbon has the ability to remove impurities, its primary drawback is that it is costly and difficult to renew, which drives up the cost of wastewater treatment. Therefore, in order to make the adsorption process economically viable, it is critical to choose inexpensive adsorbents that do not require additional pretreatment steps. Because of its immunogenicity, biodegradability, and gel formation capability, alginate is a chemical that is considered to be of great value.

In the domain of environmental science, alginate beads have shown effectivity in removing ions of certain heavy metals and dyes^19^. In neutral and alkaline conditions, this polysaccharide’s carboxylate functional groups are negatively charged and have a larger affinity for cations. For instance, Pandey et al.^20^ utilized ca-alginate beads to remove metal ions whereas, Jang et al.^21^ applied sodium alginate in copper bioremoval; on the other hand, Min, and Hering^22^ removed Ar, Cr and Se from wastewater using alginate. Additionally, several alginate gels were studied by Hyun Gyu Park^23^ in order to remove of lead (II): formation of a thin layer of alginate on an inert matrix yields alginate gel-coated adsorbent and alginate beads (paper or cotton). In addition, it has been demonstrated that alginic acid is effective in eliminating a wide variety of cationic metals from solutions, such as pb, Cu, Cd, Zn, and Co^24^. Additionally, there have been studies regarding the employment of alginate beads for the removal of organic pollutants, as stated by Rocher et al.^19^ who used ca-alginate beads to bioremove colors from commercial tannery effluents. Moreover, adsorption of activated carbon fiber, modified alginate, and basic dyes was evaluated by Nasr et al.^25^.

The main objective of the current study is evaluation of the potentiality of alginate beads derived from *Padina pavonica* for crystal violet biosorption from wastewater, their adsorption properties were evaluated through adsorption isotherms and kinetics studies. Environmental factors effect on biosorption as pH, contact time, crystal violet concentration and alginate dose were investigated.

## Materials and methods

### Collection of *Padina pavonica*, preparation, extraction and characterization of sodium alginate

This section was performed and published in a previous article^26^. *Padina pavonica* was collected from of Abu-Quir coast – Alexandria – Egypt. It was washed thoroughly several times to remove all impurities, dried and milled. Sodium alginate was extracted, dried and characterized, then it was ready to be utilized in the current study.

### Alginate beads preparation

The protocols outlined by Mandal et al.^27^ were followed in order to prepare the alginate beads. Using a glass syringe, 100 mL of sodium alginate solution with a concentration of 2% was dropped on 200 mL of CaCl_2_ solution (3%), and the mixture was agitated at 400 RPM for a duration of 1 h. Filtered beads were gathered and then washed with distilled water.

### Crystal violet solution preparation

Crystal violet (Supplementary 1) is an organic chloride salt that is the monochloride salt of crystal violet cation. To produce a crystal violet stock solution, 500 mg was dissolved in 1000 ml of distilled water. It was preserved in dark bottle in 4 °C and utilized in all experiments of the study.

### Factors affecting biosorption of crystal violet on alginate beads

#### Zero point charge

A series of flasks was prepared, each containing 25 ML of distilled water. To get the starting pH values (pHi) between 2 and 11, 0.1M HCl or 0.1M NaOH were utilized. Every flask had 50 mg of calcium alginate beads added to it and stirred at 35 ± 0.05 °C for 24 h until it reached equilibrium. After filtering the solutions, we determined their equilibrium pH levels, denoted pH_e_, plotted to obtain zero point charge.

### Initial pH

Initial pH ranges between 4 and 11 at 35 °C were investigated for their impact on the biosorption of CV pollution dye (10 mg L^−1^). The solutions (25 mL) were supplied with 50 mg calcium alginate beads, and they were agitated for 7 h at 150 rpm. The change in dye concentration was estimated by spectrophotometer. The adsorbed CV concentration was calculated by Eq. (1):

1 $${\text{Q}}_{{\text{t}}} = {\text{ V }}\left( {{\text{C}}_{{\text{o}}} - {\text{ C}}_{{\text{t}}} } \right) \, /{\text{m}}$$

where CV volume is expressed as V(L), initial concentration of CV is expressed as C_o_ (mg L^−1^), final concentration is expressed as C_t_ (mg L^−1^), and alginate bead weight is expressed as m (g).

### Incubation time

Biosorption efficiency of Crystal violet pollutant dye was investigated using 25 ml of CV solution (10 mg L^−1^) at pH 6 with 50 mg of calcium alginate. At intervals of one to 7 h, bottles were rotated at 150 rpm and 35 °C.

### Kinetic studies

#### pseudo-first order kinetic model

The equilibrium between the solution and the biosorption is established by the reversible linear process of ion biosorption from an aqueous solution onto adsorbents. Lagergren’s Equation^28^ is expressed as.

2 $${\text{Log }}\left( {{\text{Q}}_{{\text{e}}} - {\text{ Q}}_{{\text{t}}} } \right) \, = {\text{ log Q}}_{{\text{e}}} - {\text{ k}}_{{1}} {\text{t}}/ \, \left( {{2}.{3}0{3}} \right)$$

where k_1_ is the pseudo-first order rate constant (min^−1^). Dye biosorption at equilibrium and time (t) are Q_e_ and Q_t_ (mg g^−1^), respectively.

#### pseudo-second order kinetic model

The formula for pseudo-second order kinetics is generally employed in the form proposed by Kang et al.^29^ as,

3 $${\text{t}}/{\text{Q}}_{{\text{t}}} = { 1 }/ \, \left( {{\text{k}}_{{2}} {\text{Q}}_{{\text{e}}}^{{2}} } \right) \, + \, \left( {{1 }/{\text{ Q}}_{{\text{e}}} } \right){\text{ t}}$$

the pseudo-second order rate constant of biosorption is denoted by k_2_, Q_e_ and Qt are used to express the quantity of dye biosorption that occurs at equilibrium and at time (t), respectively. Both the fitness of the straight line (R^2^) and the consistency between the values of Q_e_ that were calculated and those that were observed in the experiments are used to determine whether or not a model is valid.

### Intraparticle diffusion model

The intraparticle diffusion model, may be explained by applying the Weber–Morris Equation^30^

4 $${\text{Q}}_{{\text{t}}} = {\text{ k}}_{{{\text{int}}}} {\text{t}}^{\raise.5ex\hbox{$\scriptstyle 1$}\kern-.1em/ \kern-.15em\lower.25ex\hbox{$\scriptstyle 2$} } + {\text{C}}_{{\text{i}}}$$

where k_int_, intraparticle diffusion rate constant (mg g^−1^ min^1/2^) which characterizes the boundary layer thickness.

### Elovich model

The rate of chemical processes where gases are adsorbed onto a solid surface without the products being desorption slows down with time as the surface coverage increases. One of the most beneficial models for characterizing the adsorption of activated chemicals is the Elovich kinetic equation.

5 $${\text{Q}}_{{\text{t}}} = { 1}/{\text{b ln}}\left( {{\text{ab}}} \right) + { 1}/{\text{b ln t}}$$

The initial adsorption rate is given by a (mg g^−1^ min^−1^), and b (g mg^−1^) is linked to the surface area and the activation energy for chemisorption. When ln t = 0, (1/b) and (1/b) ln (ab) indicate the number of adsorption sites and adsorption quantity.

### Alginate beads dose

Alginate dosage influence was investigated by applying alginate doses in the range from 28 to 140 mg, CV concentration of 10 mg L^−1^ (25 mL) at the optimum pH level, and stirring at 150 rpm for 5 h at 35 °C.

### Crystal violet concentration

In order to investigate CV biosorption on calcium alginate beads, the initial CV concentration (mg L^−1^) was varied from 2 to 20 mg L^−1^ for 5 h at 35 °C and pH 6.

### Adsorption isotherm models

The data obtained has been fitted to the four widely accepted adsorption isotherms, Langmuir^31^, Freundlich^32^, Dubinin–Radushkevich (D-R) isotherm models^33 and Redlich-Peterson isotherm model^.

### Langmuir Isotherm model

The Langmuir isotherm describes the process by which a homogeneous monolayer of adsorbate deposits itself on the outer adsorbent surface until it reaches saturation. The formation of a monolayer on a handful of adsorption sites that are identical can be accomplished successfully with the help of this isotherm. The Langmuir isotherm model’s linear Eq. (6) is presented in the following:

6 $${1}/{\text{Q}}_{{\text{e}}} = { 1}/{\text{Q}}_{{\text{m}}} + { 1 }/ \, \left( {{\text{Q}}_{{\text{m}}} {\text{K}}_{{\text{L}}} {\text{C}}_{{\text{e}}} } \right)$$

Q_e_ represents the quantity of adsorbate that is present on the adsorbent when it is in a state of equilibrium (mg g^−1^), C_e_ represents the equilibrium concentration (mg L^−1^), Q_m_ represents the maximum adsorption capacity (mg g^−1^), and K_L_ represents the Langmuir constant (L mg^−1^).

### Freundlich isotherm

The adsorbate forms several layers on adsorbent surface, as revealed by the Freundlich model, This can be expressed by Eq. (7).

7 $${\text{log Q}}_{{\text{e}}} = {\text{ log K}}_{{\text{F}}} + {1}/{\text{n }}\left( {{\text{log C}}_{{\text{e}}} } \right)$$

The adsorption efficiency, which is affected by the material’s heterogeneity, is shown by the Freundlich constants n and K_F_.

### Dubinin–Radushkevich isotherm model

Dubinin–Radushkevich (D–R) isotherm model shows the mechanism of the adsorption as chemical, physical, or ion exchange. Equation (8) represents the adsorption of energy

8 $${\text{Ln Q}}_{{\text{e}}} = {\text{ln Q}}_{{\text{s}}} {-} \, \beta \, \varepsilon^{{2}}$$

In this equation, Qe (mg g^−1^) indicates CV adsorption onto alginate beads, The constant β represents the average adsorption free energy calculated per mole of adsorbate, ε is the Polanyi potential, and Qs (mg g^−1^) is the maximum adsorption capacity. Equations (9) and (10) will be used to illustrate these concepts.

9 $$\varepsilon \, = {\text{ RTln }}\left( {{1} + {1}/{\text{C}}_{{\text{e}}} } \right)$$

10 $${\text{E}} = { 1}/\left( {{2}\beta } \right)^{{{1}/{2}}}$$

T is the absolute temperature (K); ε is the Polanyi potential; R is the universal gas constant (8.314 J mol^−1^), C_e_ is solution concentration at equilibrium, E is energy (kJ mol^−1^), β is constant related to the mean free energy of adsorption per mole of adsorbate.

### Redlich-Peterson isotherm model

In order to address the Freundlich isotherm argument that could not be reduced to Henry’s Law at low concentration, the Redlich-Peterson isotherm was established by combining the Freundlich and Langmuir isotherms. The equation below represents the isotherm:

11 $${\text{Q}}_{{\text{e}}} = {\text{ K}}_{{\text{R}}} {\text{C}}_{{\text{e}}} / \, \left( {{1} + {\text{a}}_{{\text{R}}} {\text{C}}_{{\text{e}}} } \right)^{{{\text{bR}}}}$$

where K_R_, a_R_ and b_R_ are Redlich-Peterson isotherm constants.

### Alginate beads characterization

#### FTIR spectroscopy

Fourier transform infrared (FTIR) spectroscopy of dried alginate beads both prior to and following the process of CV bioremoval was performed within the 400–4000 cm^−1^ range.

#### Scanning Electron Microscopy (SEM)

Electron microscope was utilized to scan the alginate beads both before and after they were loaded with crystal violet.

## Results and discussion

### Zero point charge

The pH_ZPC_ value is obtained from the plot of pH_i_ vs pH_e_ by batch equilibration. The biosorbent’s zero point charge (pHₚzc) was determined to be 9, suggesting that the surface has a net positive charge at pH values below 9 and a net negative charge above this pH. Curiously, it was found that the optimal pH for crystal violet biosorption was 6 that is a pH lower than the pHₚzc. Although this may appear contradictory at first, there are a number of reasons why this behavior occurs. The biosorbent’s surface has a small positive charge at pH 6, which normally prevents the cationic dye from adhering because of electrostatic repulsion. However, biosorption is not governed only by electrostatic force. Despite surface charge effects, functional groups can still participate in hydrophobic interactions, hydrogen bonding, and π–π interactions with the aromatic rings of crystal violet so promoting adsorption^34^. Furthermore, at elevated pH levels (> pHₚzc), while the surface acquires a negative charge and electrostatic attraction is enhanced, factors such as precipitation, competition with OH^−^ ions, or dye aggregation may diminish biosorption efficiency^35^. Thus, pH 6 likely represents a balance between sufficient solubility of the dye and active functional group availability on the biosorbent surface, leading to the observed optimum biosorption. Comparable results have been documented in previous research, where optimal dye absorption proceeded below the biosorbent’s pHₚzc, suggesting the participation of non-electrostatic interactions^36^.

### Initial pH

**Fig. 1:**
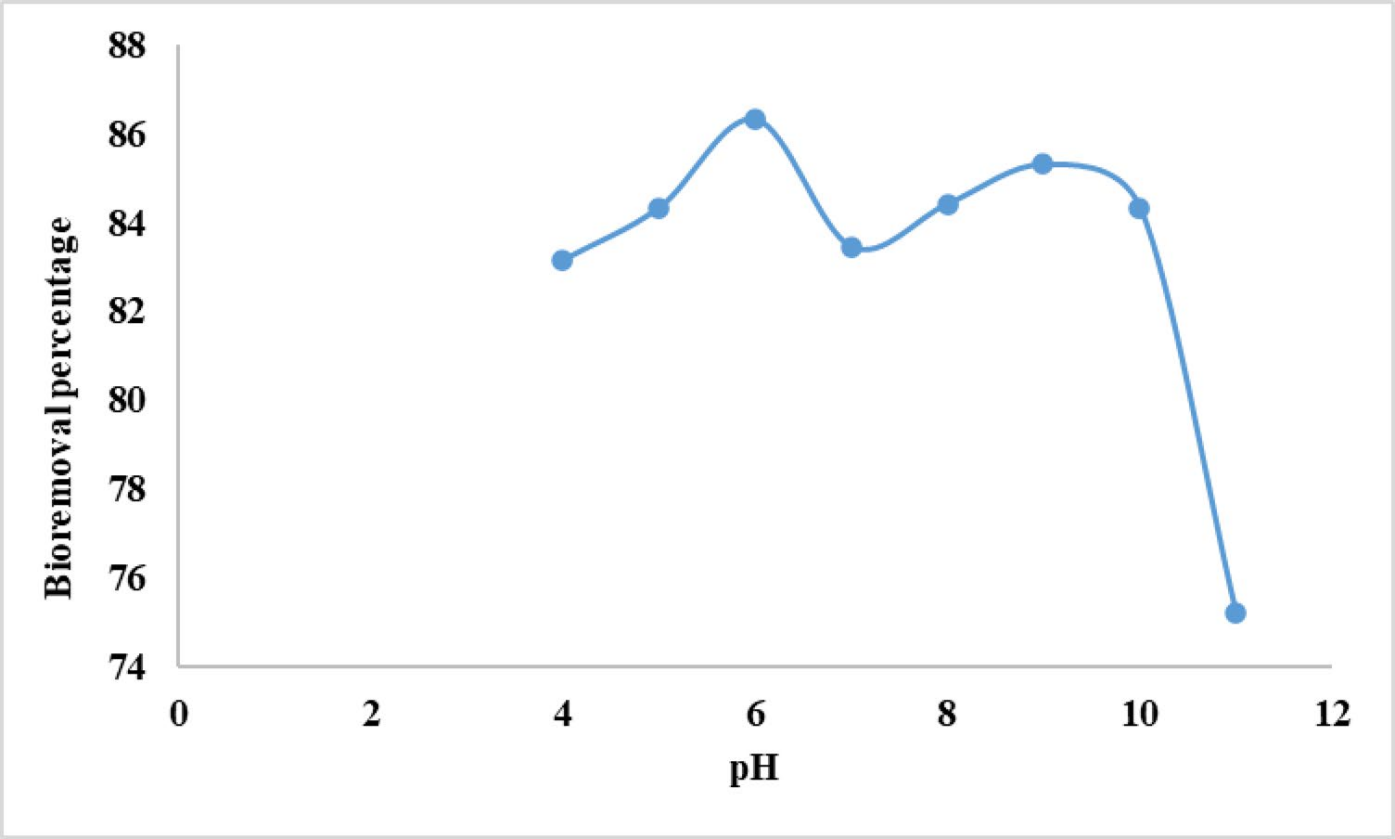
Effect of pH on bioremoval efficiency of CV from water by alginate beads at the optimum conditions temperature (35 °C), dosage of alginate biosorbant (50 mg), CV dye concentration of 10 mg L^−1^ and contact time (7 h).

The pH is a key variable in adsorption research because it influences adsorption efficiency as well as color and solubility of dyes in solutions. It influences the dye’s chemical characteristics and the active sites on the biosorbent surface^37^. The effect of pH on the adsorption of crystal violet dye by alginate beads was determined at different pH ranges (4–11). The quantities of electrostatic charges that the ionized dye molecules deliver will depend on the pH of the medium. In solutions of various pH, functional groups on both the adsorbent and adsorbate can be protonated or deprotonated to produce distinct surface charges, causing electrostatic attraction or repulsion^38^. The variation in the percentage of CV removal with pH is shown in Fig. 1. Figure 1 shows that dye removal increases slightly with increasing pH up to 6.0, then drops, concluding that pH 6 is the optimum for CV biosorption from aquatic solutions. At lower pH, protons may compete with positively charged CV dye molecules for adsorption sites, reducing dye absorption and adsorptive ability. As pH increased to 6, CV adsorption onto alginate beads increased due to dye cationic species electrostatically interacting with the negatively charged surface of alginate beads^39^. Further pH increase caused many hydroxyl ions to connect directly to positively charged dye molecules, limiting their adsorption onto adsorbent sites^40^. In accordance with our results the researchers reported that there is a significant relationship between the pH and the dye removal efficiency, and it has been stated that the ideal pH range for dye removal is located within 6 and 10 range^41^. In line with the current information, El-Naggar et al. ^42^ showed that the maximum biosorption of congo Red on Ulva lactuca from aquatic solutions was reached at pH 6. Moreover, Onuk et al.^43^ reported that the optimum biosorption of MB and CV from aquatic solutions was achieved at pH 7.

### Incubation time

**Fig. 2:**
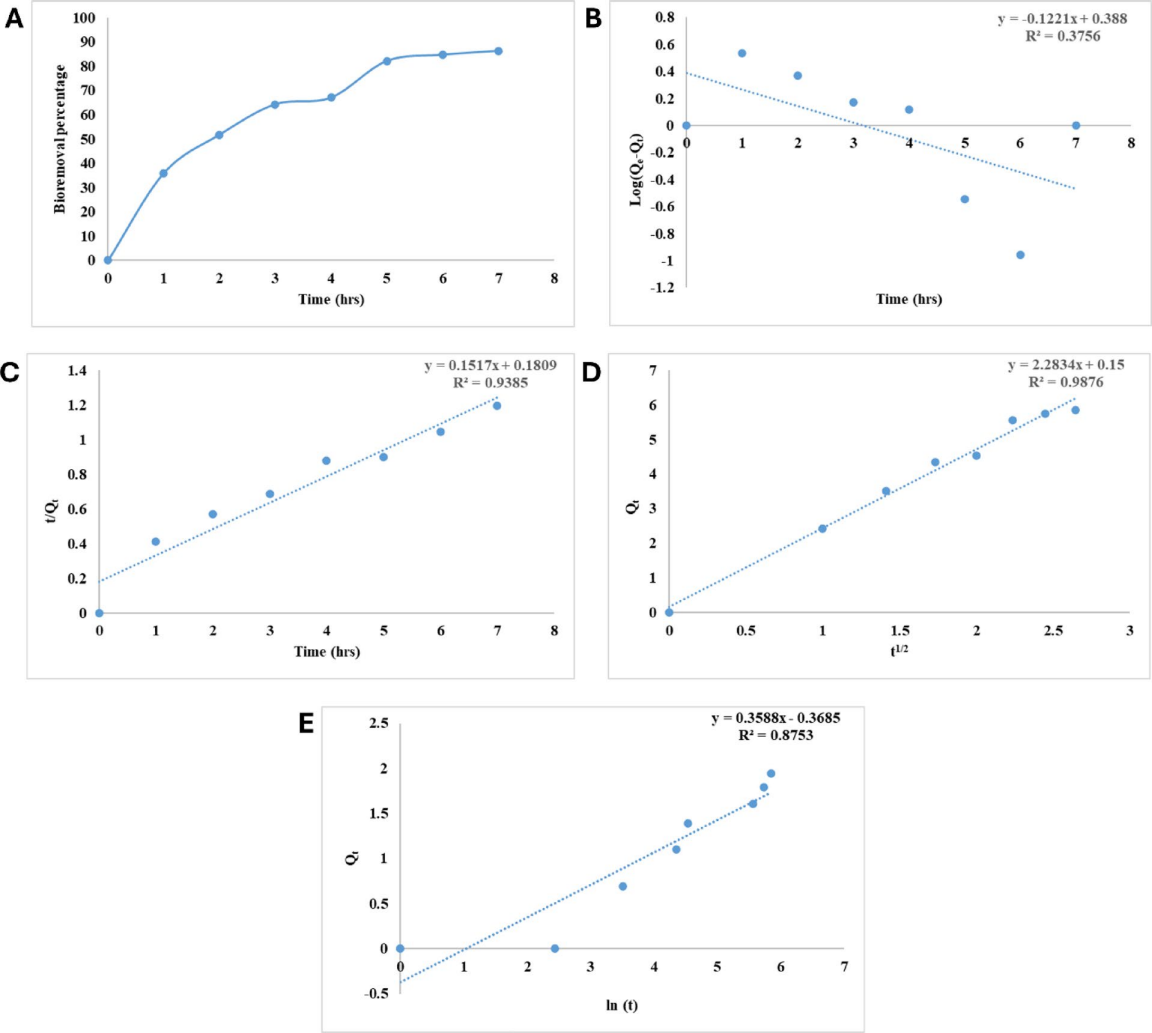
(**A**) Effect of incubation time on bioremoval efficiency of CV from water by alginate beads at the optimum conditions temperature (35 °C), dosage of alginate biosorbant (50 mg), CV dye concentration of 10 mg L^−1^, (**B**) Pseudo-first order kinetic model for CV bioremoval on alginate beads, (**C**) Pseudo-second order kinetic model for CV bioremoval on alginate beads, (**D**) Intraparticle diffusion model for CV bioremoval on alginate beads and (E) Elovich kinetic model for CV bioremoval on alginate beads.

The most critical design parameter affecting adsorption process performance is adsorbate-adsorbent contact time. In the current study, crystal violet dye bioremoval by alginate beads was evaluated at different time intervals. The incubation time effect on the capacity of CV biosorption onto alginate beads is presented in Fig. 2A. The initial biosorption rate was considerable, but it gradually decreased until it reached equilibrium at 7 h, it can be attributed to the decrease in the thickness of the diffusion layer surrounding the alginate particles^44^. Moreover, other mechanisms like binding site saturation, complexity, and microprecipitation may occur during the slower phase of biosorption^45^. The results reveal that there is a strong electrostatic force of attraction as well as a high affinity between the functional groups that are present on the alginate beads and the CV dye^46^.

In order to determine the kinetic behavior of CV biosortion by alginate beads, the experimental data were analyzed by the pseudo-first order, pseudo-second order and intraparticle diffusion kinetic models. Table 1 shows the parameters and correlation coefficients (R^2^) for pseudo-first order, pseudo- second order and and intraparticle diffusion models. pseudo-first order kinetic model data (Fig. 2B) did not fit the present results due to the great difference between the theoritical Q_e_ (1.474) and the experimental value (5.884) as well as the value of R^2^ (0.376) which was close to zero. Moreover, The pseudo-second order model provided a more satisfactory explanation for the experimental findings (Fig. 2C), and it also had a great correlation coefficient (0.939) which was found closer to unity. In addition, the experimental Q_e_ values (5.884) and the computed Q_e_ values (6.592) were compatible. Hence, the results point to a process known as chemisorption adsorption^47^. Adsorption occurs in several steps, including the Moving of CV molecules from the liquid to the alginate beads’ surface and the transfer of CV molecules from the surface of the beads into the pores of the alginate^48^.

**Table 1:** Pseudo-first order, Pseudo-second order, intraparticle diffusion kinetic models parameters for CV bioremoval on alginate beads.

Pseudo-first order kinetics			Pseudo-second order kinetics			Intraparticle diffusion			Elovich model		
R^2^	K_1_	Q_e_ _Theoritical_	R^2^	K_2_	Q_e_ _Theoritical_	R^2^	K_2_	Q_i_	a	b	R^2^
0.3756	0.281	1.474	0.939	0.127	6.592	0.988	2.283	0.15	0.338	2.958	0.857

The biosorption of crystal violet onto alginate beads in the present study reached equilibrium at 7 h and followed pseudo-second-order kinetics, indicating that chemisorption was the dominant mechanism. These findings are consistent with several previous studies. For instance, Mittal et al.^49^ observed similar equilibrium times (6–8 h) for crystal violet adsorption onto bottom ash and de-oiled soya, with data fitting well to a pseudo-second-order model. Likewise, Ismail et al.^50^ reported maximum uptake around 7 h using *Euphorbia antiquorum*-activated carbon. In contrast, other studies have shown significantly different results. Hameed and Ahmad^51^ reported a much faster equilibrium time of 2 h using bamboo-based activated carbon, attributed to the high surface area and porosity of the material. Annadurai et al.^52^ found that chitin could remove crystal violet within just 1 h. These discrepancies highlight the influence of biosorbent type, surface characteristics, and binding site accessibility on adsorption behavior.

Intraparticle diffusion model of Weber and Morris is typically utilized in order to shed light on the adsorption mechanism process that is involved^53^. When plotting Q_t_ against t^1/2^, Weber and Morris models predict that a straight line would be produced. This is an indication that the adsorption process is mostly controlled by intraparticle diffusion, the straight line is expected. Film diffusion or pore diffusion are two possible examples of the rate-limiting steps^54^. By interpreting Q_t_ versus t^1/2^, (Fig. 2D) a linear form was obtained, which indicates the occurrence of intraparticle diffusion^55^. Finding the slope of the linear section of the kinetics profile yielded the value of K_int_, which stands for the intraparticle diffusion constant, which is 2.283, while C_i_ was obtained from the intercept and its value (0.15 mg g^−1^), provides border thickness information layers as reported by Saad et al.^56^.

Elovich’s model of activated chemisorption is extremely remarkable. Figure 2E depicts the Elovich model for CV adsorption on alginate beads. From plot intercept and slope, a and b values were computed and displayed in Table 1. The correlation coefficient (R^2^ = 0.875) was lower than pseudo-second-order model.

### Biosorbent dosage

**Fig. 3:**
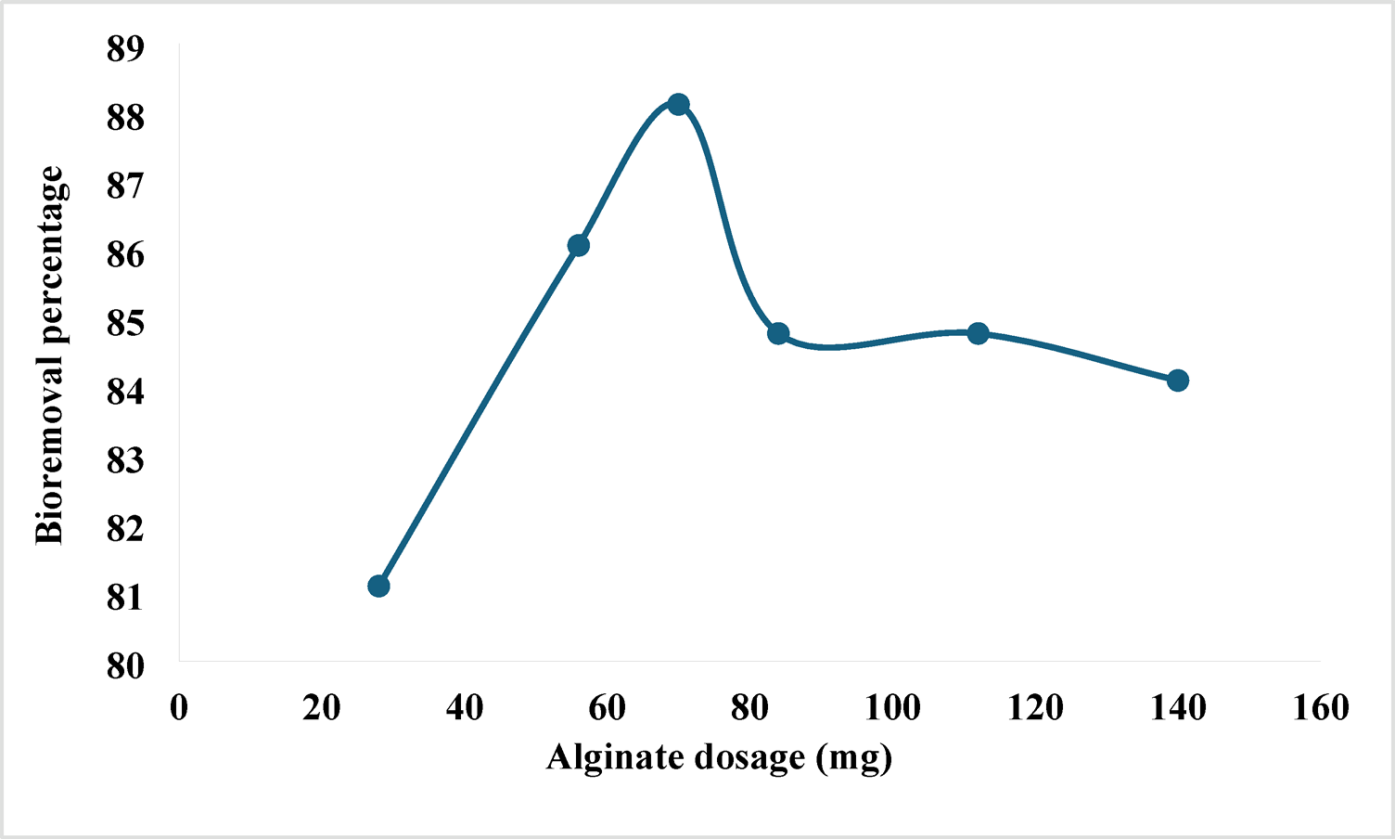
Effect of alginate beads dosage on bioremoval efficiency of CV from water by alginate beads at the optimum conditions of pH (6.0), temperature (35°C), 10 mg/L CV concentration, and contact time (5 h).

A further critical variable in determining the system’s sorbent-sorbate equilibrium for efficient pollutant dye removal is the initial adsorbent concentration. For the most effective interactions between the sorbate molecules and the sorbent’s adsorption sites in solution, it is necessary to use the appropriate amount of sorbent^57^. The effect of different calcium alginate beads dosages (28, 56, 70, 84, 112 and 140 mg/ 25 mL) on the biosorption of CV dye from the solution was investigated (Fig. 3). The results reveal that the removal of dye increased with increasing weight of alginate beads up to 70 mg because of the increase in available active adsorption sites number. The maximum dye removal efficiency (88.1%) was obtained by 70 mg of alginate beads. Due to particle aggregation^58^, or insufficient CV in the solution^59^, increasing alginate dosage did not promote increments in dye removal. Moreover, higher dose of biosorbent may result in formation of agglomerates, which in turn reduces the effective surface area that dye molecules have access to^60^.

In the current study, the biosorption capacity of alginate beads for crystal violet increased with increasing biosorbent dose, reaching optimal removal at 70 mg. This trend is consistent with previous research, where increasing biosorbent dosage enhanced dye removal due to the greater availability of active binding sites. For instance, Mittal et al.^61^ reported a similar positive correlation when using de-oiled soya and bottom ash for crystal violet removal, observing improved adsorption efficiency with increasing sorbent mass up to a threshold. Likewise, Annadurai et al.^52^ found that higher doses of chitin significantly increased dye uptake, likely due to reduced competition among dye molecules for sorption sites.

However, some studies have noted a plateau or even a decline in adsorption efficiency at higher doses. Hameed and Ahmad^51^ using bamboo-based activated carbon, observed that after a certain point, further increases in sorbent mass did not significantly improve removal efficiency, possibly due to particle aggregation, which reduces the effective surface area. Similarly, Ismail et al.^50^ found that excess biomass might lead to site overlapping or blockage, lowering the apparent adsorption per unit mass.

These findings suggest that while increasing biosorbent dose generally enhances removal efficiency, there exists an optimal dosage such as the 70 mg found in this study beyond which the biosorption may plateau or diminish, depending on the biosorbent’s structure and interaction dynamics with the dye.

### Crystal violet initial concentration

**Fig. 4:**
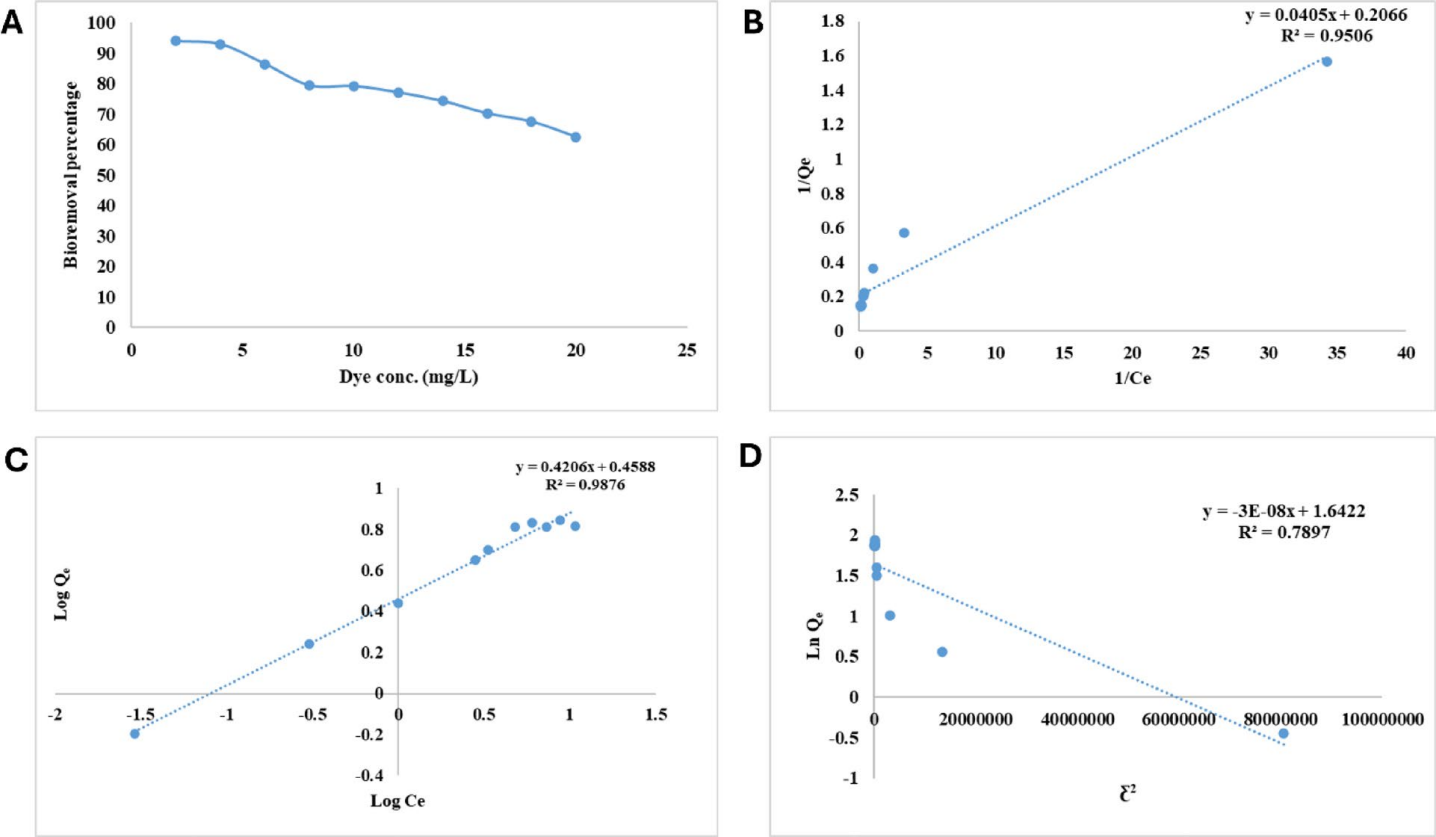
(**A**) Effect of initial CV concentration on bioremoval efficiency of CV from water by alginate beads at the optimum conditions of pH (6.0), temperature (35 °C), dosage of alginate biosorbant (70 mg), and contact time (5 h), (**B**) Langmuir isotherm for CV bioremoval on alginate beads, (**C**) Freundlich isotherm for CV bioremoval on alginate beads and (**D**) Dubinin–Radushkevich isotherm for CV bioremoval on alginate beads.

The initial concentration of the dye is also another significant factor that has the potential to influence the adsorption process. Dye concentrations (2–20 mg L^−1^) varying impact on CV biosorption at 35 °C, and the optimum conditions of pH (6.0), dosage of alginate biosorbant (70 mg), and contact time (5 h) is illustrated in Fig. 4A. Results demonstrated that as dye concentration increased, the efficacy of alginate beads in removing CV pollutant dye declined (Fig. 4A). The maximum bioremoval percentage of CV dye (93.917%) was attributed to the lowest dye concentration (2 mg L^−1^). Once the dye concentration rises to 10 mg L^−1^ the CV bioremoval percentage decreased dramatically to 62.58% indicating overload of the alginate beads active binding sites. Biosorption experimental data are usually approximated as equilibrium isotherms, which give important parameters for mechanism prediction and process optimization^62^.

In the present study, the biosorption efficiency of crystal violet by alginate beads decreased with increasing initial dye concentration. Similar observations were reported by Chandra et al.^49^ who found that increasing the concentration of crystal violet led to reduced removal efficiency when using waste materials such as de-oiled soya and bottom ash. Likewise, Annadurai et al.^52^ reported that higher concentrations of methylene blue and reactive dyes decreased biosorption by chitin, suggesting that at elevated concentrations, dye molecules compete more intensely for limited binding sites. On the other hand, some studies have reported a different trend. For example, Hameed and Ahmad^51^ observed that the adsorption capacity (mg g^−1^) of bamboo-based activated carbon increased with rising dye concentration, although the percentage removal decreased, indicating that total uptake may still improve despite lower efficiency. Similarly, Ismail et al.^50^ found that higher initial dye concentrations provided a greater driving force for mass transfer, which enhanced the biosorption rate in the early stages. These discrepancies highlight the importance of distinguishing between percentage removal and adsorption capacity, and they underline the role of biosorbent surface characteristics and saturation kinetics.

The Langmuir, Freundlich, and Doubinin–Radushkevich isotherms models were utilized in order to accomplish the evaluation of the biosorption of CV onto alginate beads. Due to its ease of use and high degree of concordance with the experimental data, Langmuir model is considered to be one of the most prevalent and commonly utilized models for equilibrium data^63^. The Langmuir isotherm model assumes biosorption occurs in monolayers at homogeneous biosorbent sites^64^. The results that were applied to the Langmuir isotherm model did not come close to matching the results of the experiments. Based on the plot of 1/Q_e_ vs 1/C_e_, which can be found in Fig. 4B, the values of K_L_, Q_m_, and R^2^ are 5.101 mL mg^−1^, 4.08 mg g^−1^, and 0.951, respectively (Table 2).

**Table 2:** Langmuir, Freundlich and Dubinin–Radushkevich isotherms parameters for CV bioremoval on alginate beads.

Langmuir			Freundlich			Dubinin–Radushkevich				
R^2^	Q_m_	K_L_	R^2^	K_f_	n	R^2^	Q_s_	β	E	Reaction type
0.951	4.084	5.101	0.988	1.582	2.378	0.79	5.167	3*10^–8^	4082.482	chemical

The dimensionless constant R_L_ can be used to evaluate Langmuir biosorption reaction processes:

10 $${\text{R}}_{{\text{L}}} = {1}/\left( {{1} + {\text{K}}_{{\text{L}}} {\text{C}}_{{\text{o}}} } \right)$$

R_L_ values indicate bisorption process favorability (0 < R_L_ < 1), unfavorability (R_L_ > 1), linearity (R_L_ = 1), or irreversibility (R_L_ = 0)^37^. The value of the R_L_ constant for the biosorption of CV by alginate beads was 0.871, which indicates that the biosorption process is favorable.

For the adsorption of CV dye on alginate beads, the Freundlich isotherm demonstrated that the plot of log Q_e_ vs log C_e_ results in a straight line with a slope of 1/n and an intercept of ln K_F_ (Fig. 4C). The values of Freundlich constant (K_F_), correlation coefficient (R^2^) and n were 1.582 mg g^−1^, 0.988 and 2.378, respectively (Table 2). The value of n less than 10 revealing a favorable adsorption^65^. Sum of squares Error was calculated according to the experimental data and the calculated data, giving 0.501, whereas Root Mean Square Error was 0.708. in addition, Akaike criterion information was found to be 2.473. Since the adsorption capacity is proportional to the value of K_F_ as reported by Ibrahim et al.^66^ so, K_F_ value of CV biosorption on alginate beads indicates high adsorption capacity. According to the findings that we obtained; the process is consistent with the Freundlich isotherm model. Furthermore, alginate beads surface is regarded to be heterogeneous, and the CV is arranged in multilayers on the surface of the alginate beads^32^.

The Dubinin–Radushkevich model (Fig. 4D) describes the Gaussian energy distribution adsorption mechanism on a heterogeneous surface^67^ and is broader than is the Langmuir model. Physical adsorption occurs when E is below 8 kJ mol^–1^. On the other hand, ion exchange takes place when E ranges from 8 to16 kJ mol^–1^^68^. From Table 2, it is obvious that energy value was more than 16 so it is concluded that the effect of chemical adsorption will play a dominating role in the adsorption of CV onto alginate beads. A comparative table (Table 3) of CV biosorption of biosorbents is provided^69–71^.

**Table 3:** A comparative table of CV biosorption of biosorbents.

Biosorbent	Q_max_ (mg/g)	References
*Equisetum ramosissimum*	17.27	^9^
*Citrullus lanatus*	46.68	^10^
*Cyperus rotundus*	54.24	^10^
Kaolinite mixed with cellulose from red bean peels	294.12	^11^

Empirical Redlich–Peterson isotherm model has three parameters. It represents adsorption equilibrium across a wide range of adsorbate concentration in homogenous or heterogeneous systems by combining Langmuir and Freundlich Equations ^72^. Table 3 displays the estimated parameters (KR, Redlich-Peterson isotherm constant (Lg − 1), aR, and bR) using the three-parameter isotherm model to determine the greatest correlation coefficient. R^2^ value of unity was obtained at K_R_ of 2.874 Lg^−1^ for 8 mg L^−1^ to 20 mg L^−1^ crystal violet initial concentration, demonstrating the ease of adsorption as the initial CV concentration rises. Redlich*–*Peterson approaches the Freundlich isotherm model as *b*_R_ tends to be less than 1 supporting multilayer adsorption^73^. Concluding that, the most *b*R values were less than 1 (Table 4) suggest that this Freundlich model best describes CV adsorption on alginate beads.

**Table 4:** Redlich–Peterson isotherms parameters for CV bioremoval on alginate beads.

Initial dye concentration	a_R_	b_R_	k_R_	R^2^
2	0.1	1	2.874	0.2997
4	0.1	1	2.874	0.4728
6	0.1	0.9	2.874	0.7783
8	0.2	0.6	2.874	0.9999
10	0.5	0.2	2.874	0.9999
12	0.7	0.4	2.874	0.9997
14	0.8	0.5	2.874	0.9999
16	0.5	0.8	2.874	0.9992
18	0.9	0.6	2.874	0.9999
20	0.5	0.9	2.874	0.9999

### Alginate beads characterization before and after CV biosorption

#### FTIR spectroscopy

**Fig. 5:**
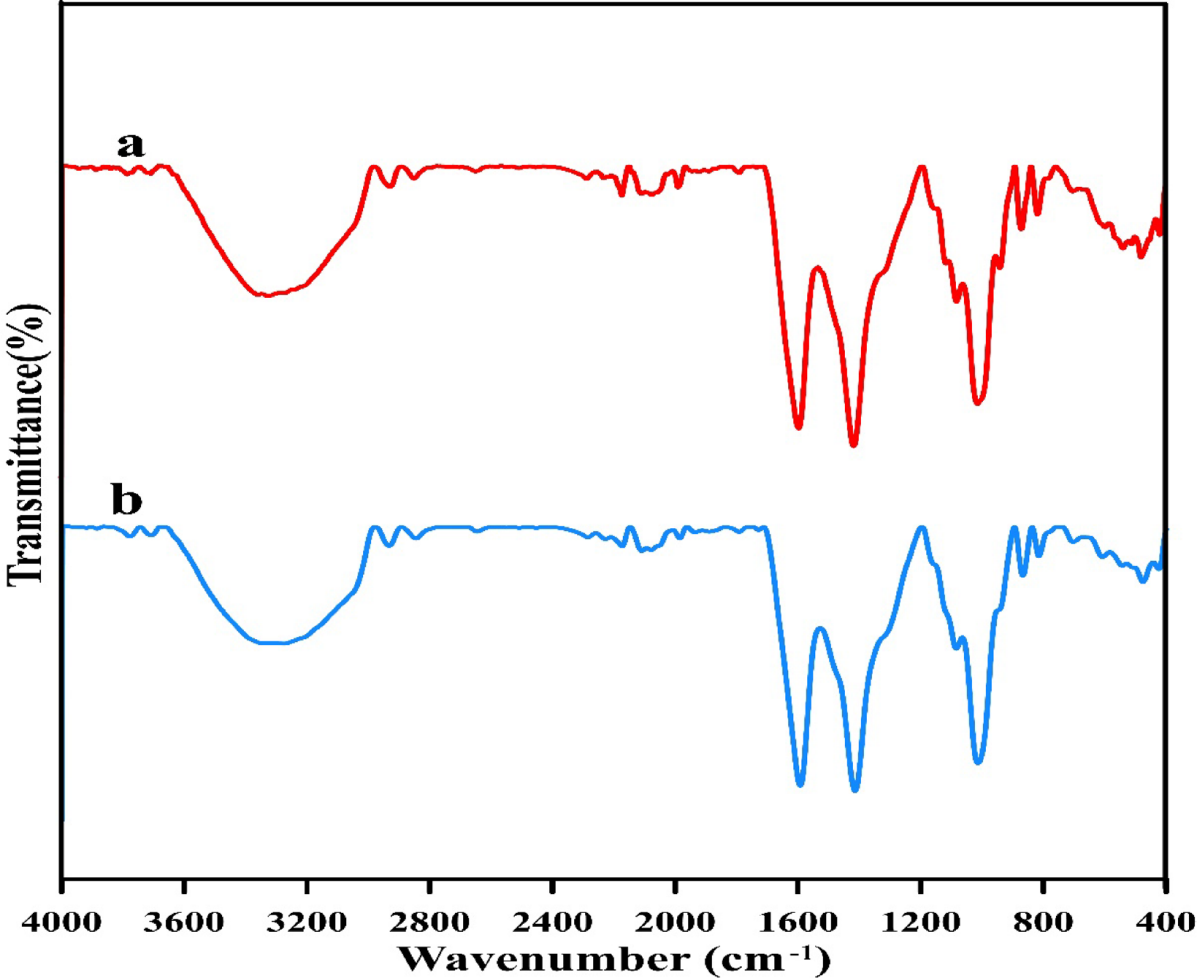
FTIR spectra of alginate beads (**a**) control beads and (**b**) beads loaded with CV dye.

The FTIR spectrums of untreated alginate beads and alginate beads loaded with CV pollutant dye were analyzed (Fig. 5) to discover changes caused by CV-alginate beads functional group interactions during biosorption. FTIR analysis of control alginate beads show different adsorption peaks at 3326, 2931, 2851, 1595, 1417, 1116, 1081, 1013, 940, 870, 817, 597, 539, 510, 580 and 419 cm^−1^ which were shifted to 3274, 2933, 2845, 1592, 1414, 1084, 1013, 867, 815, 703, 607, 543, 475 and 424 cm^−1^ after biosorption process. A strong absorption band at 3326 cm^−1^ is observed in the Fourier transform infrared (FTIR) spectrum of control alginate beads powder, which indicates the presence of hydroxyl groups as anticipated^74^. Apart from aromatic and/or vinylic C–H stretching, spectral band at 2931 cm^−1^ can be ascribed to aliphatic –CH stretching and symmetric and asymmetrical (C–H)CH_2_ stretching, (CH)-anomer stretching^75^. The absorbance at 2851 cm^−1^ represents –CH_3_ groups^76^. The spectrum band at 1995 cm^−1^ shows the stretching vibrations of carboxylate anions (COO–), which can be either asymmetric or symmetric^75^. Spectral band that located 1417 cm^−1^ is related to –N–H bending^77^. The sodium alginate polysaccharide structure is also characterized by the other peaks that arise in the region between 1190 and 900 cm^−1^. The [C–O] and [C–C] stretching vibrations are linked to these peaks^78^.

The absorption peak at 870 cm^−1^ wave number is the characteristic absorption peak of CO3 vibrations of calcite^79^. The spectral band at 940 cm^−1^ is attributed to O–H bending out of plane^80^. Alginate’s primary components, guluronic and mannuronic acids, are revealed by a spectral peak at 817 cm^−1^, giving it a distinct spectral signature that sets it apart from other polysaccharides^81^. Moreover, Khattar et al.^82^ suggested that the existence of several peaks less than 1000 cm^−1^ may be according to various visible bands and/or to the existence of some linkages among monosaccharides.

There is a shift in the wave number of the Peaks that occurs after CV biosorption, either upwards or downwards. The hydroxyl group absorption peak at 3326 cm^−1^ was shifted to 3274 cm^−1^ which is possibly due to the interaction of O–H group with COO − Na + to form carboxymethyl groups^83^. Moreover, the peaks 2931, 2851, 1595 and 1417 were shifted to 2933, 2845, 1592 and 1414 cm^−1^. Also a spectral band of 1081, 870 and 817 cm^−1^ were shifted to 1084, 867 and 815 cm^−1^. A new spectral band appeared in alginate beads loaded with CV at 703 cm^−1^ which may be attributed to C–C1 stretching^84^. Moreover, the spectral band at 597 cm^−1^ was shifted to 607 cm^−1^. The peak at 539 cm^−1^ was shifted to 543 cm^−1^ indicating asymmetric deformation vibration of P=O in PO4^3–85^. The spectral band at 480 and 419 cm^−1^ were shifted to 475 and 424 cm^−1^, respectively. Due to the fact that there was a slight difference in the number of peaks that occurred after and before the CV bioremoval, it was assumed that the dye that was present in the calcium alginate beads was interacting with the active functional groups^86^. In conclusion, the FITR analysis demonstrated that the carboxylic, methyl, and hydroxyl groups were the primary groups that were engaged in the CV biosorption process. For the CV binding, the carboxylic functional group is responsible for providing the majority of the biosorption sites^87^.

### Scan electron microscopic imaging

**Fig. 6:**
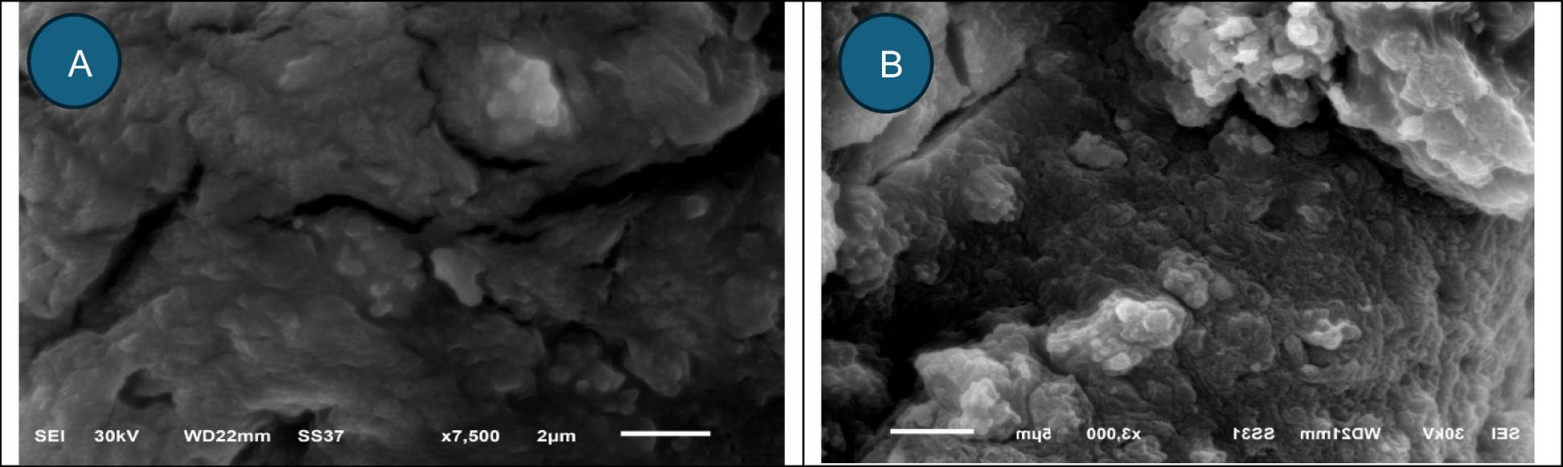
Scanning Electron Micrograph of alginate beads (**a**) control beads and (**b**) beads loaded with CV.

Alginate beads is an excellent material for CV bioremoval, was examined via SEM. Alginate beads before and after loading with crystal violet are presented in Fig. 6a,b respectively. In line with present data, Malakar et al.^88^ revealed that alginate beads possess a spherical morphology with rough external surfaces, and that the spherical configuration is typically compromised upon drying. The cross-sectional SEM images revealed many closed holes of various widths upon loading with CV.

## Conclusion

Crystal violet (CV) is a hazardous dye pollutant with significant environmental and health impacts. This study demonstrates that calcium alginate beads derived from *Padina pavonica* offer an efficient and environmentally friendly solution for CV removal from aqueous solutions. Under optimized conditions (pH 6, 70 mg/25 mL biosorbent dose, 2 mg L⁻^1^ CV concentration, and 5 h contact time), a maximum biosorption efficiency of 98.3% was achieved. Further increases in dye concentration or biosorbent dose did not significantly improve removal efficiency, suggesting saturation of available active sites. The biosorption process was best described by the pseudo-second-order kinetic model and the Freundlich isotherm, indicating multilayer adsorption on heterogeneous surfaces and chemisorption as the dominant mechanism.

Compared to conventional adsorbents like activated carbon, *Padina pavonica*-based alginate beads offer several practical advantages, including low cost, renewability, ease of preparation, and minimal environmental impact. These features support their potential scalability and real-world applicability in wastewater treatment, particularly for dye-contaminated industrial effluents. Therefore, this biosorbent presents a sustainable and cost-effective alternative to commercial materials for environmental remediation.

## Electronic supplementary material

Below is the link to the electronic supplementary material.

Supplementary Material 1

## Data Availability

All data generated or analysed during this study are included in this published article.
