# Supplementary figures and images for: Efficacious biosorption of crystal violet pollutant dye from aqueous solutions via Padina pavonica derived alginate
Source: Sci Rep. 2025 Jul 12;15:25199. doi: 10.1038/s41598-025-09752-y (PMC12255697; doi:10.1038/s41598-025-09752-y)

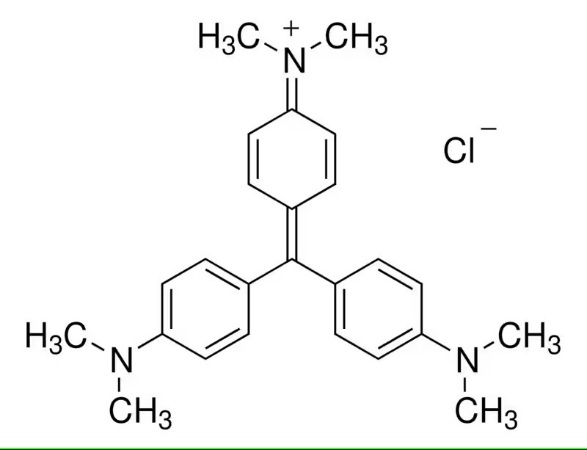


S 1: the structural formula of crystal violet

Supplement: Supplementary file 1 — Supplementary Material 1 [file 41598_2025_9752_MOESM1_ESM.docx]
